# Successful outcome of open distal interphalangeal joint luxation in equids treated by surgical debridement without internal implants

**DOI:** 10.1007/s11259-026-11487-8

**Published:** 2026-08-29

**Authors:** L. C. Ferrari, E. V. P. Apolonio, S. L. de Oliveira-Gonçalves, A. L. A. Rambo, J. V. Bergamasco, J. M. Alonso, M. J. Watanabe, A. L. G. Alves

**Affiliations:** 1 Department of Veterinary Surgery and Animal Reproduction, São Paulo State University - UNESP, Botucatu, São Paulo Brazil; 2 Department of Veterinary Medicine, Faculty of Animal Science and Food Engineering, University of São Paulo, Pirassununga, São Paulo Brazil; 3 Department of Animal Medicine, Federal University of Rio Grande do Sul, Porto Alegre, Rio Grande do Sul Brazil

**Keywords:** Conservative treatment, Horses, Joint luxation, Open wound, Surgery

## Abstract

**Introduction:**

The distal interphalangeal joint (DIPJ) plays a crucial role in the function of the hoof and digit. Dislocations and subluxations in this region are rare and primarily of traumatic origin.

**Case Report:**

This case report aims to describe two unusual cases of open DIPJ dislocation, including clinical presentation, diagnostic workup, treatment, and long-term follow-up. Both animals presented with open traumatic distal interphalangeal joint (DIPJ) luxation. In Case 1, the animal received initial care on the property 5 h post-trauma and presented with joint exposure and articular surface contact with the ground. In Case 2, the animal was referred to the veterinary hospital without prior care, 3 h post-trauma, but there was no contact of the articular surface with the ground. Both animals showed grade 5/5 lameness (AAEP). The animals underwent general anesthesia, joint lavage, regional intravenous perfusion with antibiotics, debridement of devitalized tissues, skin synthesis, and immobilization with synthetic casting. During immediate recovery and hospitalization, a grade 4/5 lameness persisted. Long-term radiographic control revealed the development of degenerative joint disease in both animals. After 3 and 2 years, respectively, animals 1 and 2 showed persistent lameness (grade 2/5) with an absence of local sensitivity.

**Conclusion:**

The conservative treatment employed in these cases of open DIPJ luxation proved effective for the maintenance of the patients’ lives, and although the infectious pathological process invariably progressed to osteoarthritis, the evolution of the clinical conditions allowed for light exercise and maintenance of reproductive activity.

## Introduction

The distal interphalangeal joint (DIPJ) plays a crucial role in the function of the hoof and digit. Changes in hoof balance, terrain variation, and multidirectional movements are accommodated and compensated through angular adjustments in the DIPJ (Clayton 2010). The DIPJ capsule, collateral ligaments, and distal sesamoid bone ligaments ensure the stability of the DIPJ against shearing and torsional forces (Zubrod and Schneider 2019). Dislocations and subluxations in this region are rare and primarily of traumatic origin. Loss of congruence between the articular surfaces occurs due to rupture of the collateral ligaments and joint capsule, which is exacerbated by exposure of articular structures to the external environment through skin discontinuity and synovial fluid leakage (McIlwraith et al. 2020). The diagnosis of DIPJ luxation is based on distal limb instability and inability to flex the digit. Radiographic examination is a crucial tool for differentiating DIPJ luxation from fractures involving the second or third phalanx (Brogniez et al. 2012).

One therapeutic option is DIPJ arthrodesis, which promotes fusion between the second and third phalanges, eliminating joint movement and resulting in pain relief (Reevs et al. 1989). However, surgical intervention presents limitations, such as challenging access to joint extremities. According to Janicek and Keegan (2007), cases with spontaneous ankylosis have a better prognosis; thus, minimal manipulation and the absence of foreign bodies, like internal implants, are recommended for better functional recovery. Septic arthritis of the DIPJ in horses most commonly arises due to external wounds or punctures at the coronary band region (Lescun et al. 2004). Deep infections within the hoof pose a therapeutic challenge, and early recognition is essential to ensure timely and effective treatment (Reevs et al. 1989).

Early recognition of septic synovitis in horses, accompanied by prompt treatment, is paramount to achieving optimal management and a favorable outcome for the affected animals. (Chan et al. 2000; Wright et al. 2003; Fraser et al. 2004; Nixon 2019). A retrospective study reported a successful outcome (survival without residual lameness) in 109 out of 150 horses (72.7%) treated with joint lavage and antibiotics for contaminated and infected synovial cavities (Pille et al. 2009). Severe or chronic infections are more difficult to treat due to vascular thrombosis, ischemia, and associated tissue necrosis (Janicek and Keegan 2007). A recent multicenter retrospective study (de Souza et al. 2024) examined admission procedures and treatment strategies for septic synovial involvement across major UK veterinary hospitals. Of the cases reviewed, 70.1% (170/240) presented wounds, 79.2% (190/240) underwent arthrocentesis, and intravenous regional perfusion was performed in 28% (66/240). Surgical techniques were employed in 97.5% (234/240) of cases; 87% (209/240) underwent joint lavage, and intra-articular antibiotic administration was performed postoperatively in 54% (130/240). The long-term survival rate was reported as excellent (89.4%).

The athletic prognosis is generally poor in cases of degenerative joint disease or septic arthritis of the DIPJ. However, horses may remain otherwise healthy and capable of living freely post-ankylosis, although mechanical lameness due to joint fusion persists. The prognosis for life is highly dependent on the severity of the joint injury; nevertheless, spontaneous ankylosis without internal implants presents the highest treatment success rates in septic DIPJ conditions (Honnas et al. 1992; Zubrod and Schneider 2005, 2019).

Open joint luxation is more frequently described in the metacarpophalangeal joint (Yovich et al. 1987; Balaam and Miller 2007; Raayat et al. 2017; Borges et al. 2020). However, only one report describes an open DIPJ dislocation with a corresponding therapeutic approach and patient outcome. This case report aims to describe two unusual cases of open DIPJ dislocation, including clinical presentation, diagnostic workup, treatment, and long-term follow-up.

## Materials and methods

### Ethical approval

The participation and data from all horses were included with the express informed consent of their respective owners.

### Case description

#### Case 1

**Fig. 1:**
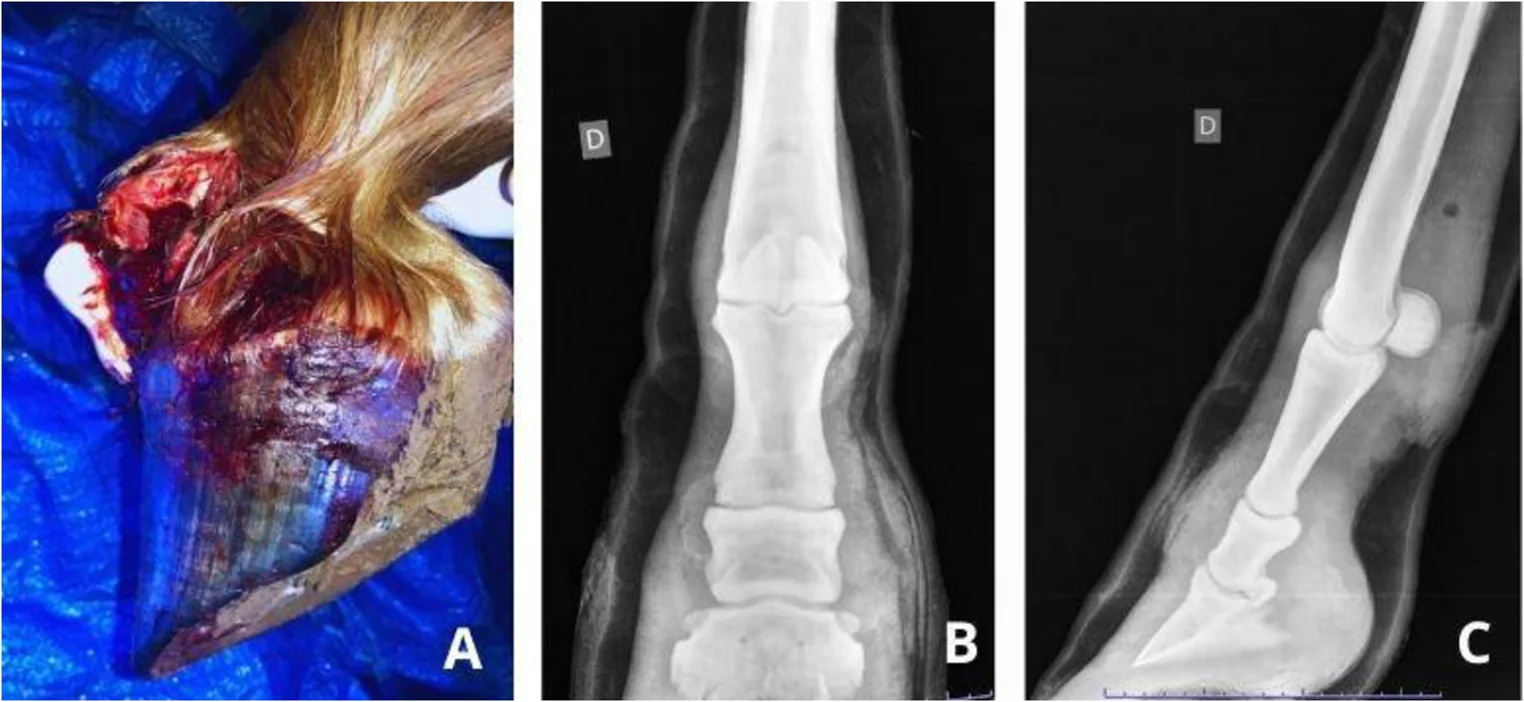
**A** Image of the dorsomedial wound with DIPJ exposure, obtained during the primary evaluation conducted on the farm. **B** Dorsopalmar view of the distal right forelimb. **C** Lateromedial view of the distal right forelimb. Radiographs were performed during the initial assessment at the veterinary hospital

A 380-kg, 6-year-old, female Quarter Horse was found suddenly in the pasture, exhibiting reluctance to ambulate, deviation of the axial alignment of the digit, and a wound with exposed bone and joint structures on the dorsomedial aspect of the right forelimb pastern. A thorough assessment of the affected area revealed complete exposure of the articular surfaces of the second and third phalanges through the wound, characterizing an open DIPJ luxation. According to the owner, the articular structures had been in contact with the ground for at least five hours before evaluation. Joint and wound decontamination were performed under intravenous general anesthesia (diazepam – 2 mg/kg, IV and ketamine; Syntec, São Paulo, Brazil – 2.2 mg/kg, IV), followed by reduction of the DIPJ dislocation (Fig. 1), and immobilization with a Robert Jones bandage and synthetic cast from the hoof to the proximal metacarpus. The horse received antibiotic and anti-inflammatory therapy (flunixin meglumine; J.A. Saúde Animal, São Paulo, Brazil – 1.1 mg/kg, IV, and ceftiofur; Vetanco, Buenos Aires, Argentina – 4 mg/kg, IM) and was referred to a veterinary hospital the next day for intensive care.

Upon hospital admission, the horse exhibited non-weight-bearing lameness (grade 5/5, AAEP). Physical examination revealed a heart rate of 72 bpm, respiratory rate of 20 bpm, rectal temperature of 38.6 °C, pink mucous membranes, and a capillary refill time of 2 s. Radiographs showed no evidence of phalangeal fractures or radiographic signs of persistent luxation (Fig. 1).

**Fig. 2:**
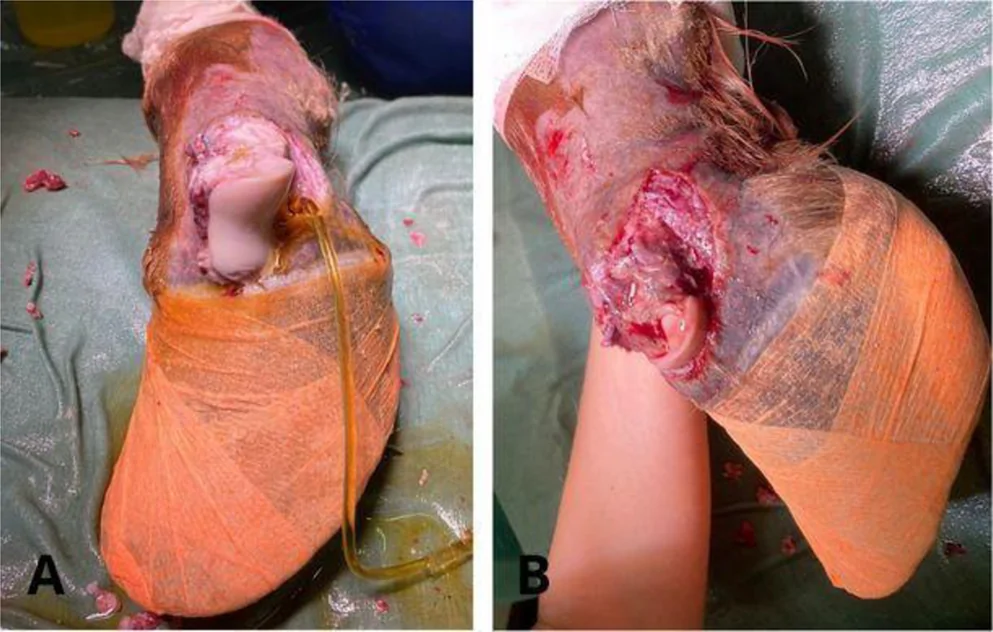
Joint re-luxation, lavage, and debridement. **A** Frontal view of the limb with complete exposition of the articular surface of the second phalange. **B** Medial visualization of the re-luxated DIPJ, highlighting medial collateral ligament rupture and the skin wound

Given the prolonged exposure and tissue contamination, the horse was placed under inhalation anesthesia. The joint was re-luxated to allow articular exposure, followed by thorough lavage with lactated Ringer’s solution and 2% iodine, and debridement of necrotic tissue while preserving the articular cartilage (Fig. 2). The coronary band skin was sutured using a simple interrupted pattern, and 150 mg of amikacin sulfate (Eurofarma, São Paulo, Brazil) was administered intra-articularly. Concurrently, regional limb perfusion (RLP) was performed using 2 g of ceftriaxone (Eurofarma, São Paulo, Brazil) diluted in 20 mL of saline and 20 mL of lidocaine hydrochloride (Cristalia, São Paulo, Brazil), with a tourniquet placed proximally on the metacarpus and venous access through the medial palmar vein.

The limb was immobilized with a 1 cm-thick polyvinyl chloride synthetic cast, incorporating an 8-cm heel elevation, extending from the hoof to the proximal metacarpus. Systemic treatment included gentamicin (6.6 mg/kg SID, IV; Syntec, São Paulo, Brazil) for 10 days, ceftiofur (5 mg/kg SID, IV) for 14 days, doxycycline (10 mg/kg BID, PO; Eurofarma, São Paulo, Brazil) for 10 days, flunixin meglumine (1.1 mg/kg SID, IV) for 4 days, a single tetanus antitoxin dose (10,000 IU, SC), and omeprazole (4 mg/kg SID; Drogavet, São Paulo, Brazil) for 30 days. On day 30, the cast was replaced with a medial splint of the same length, and after 40 days of hospitalization, the horse was discharged with grade 2/5 lameness due to reduction of limb use. The physical therapy started with hand walking was recommended.

Four months later, the horse returned with grade 4/5 lameness. Radiographs revealed narrowing of the DIPJ space, an enthesophyte on the extensor process of the third phalanx, and decreased radiopacity in the distal portion of the second phalanx, consistent with degenerative osteoarthritis. Due to persistent lameness and local pain, intra-articular injection of methylprednisolone (40 mg; Eurofarma, São Paulo, Brazil), amikacin (125 mg), and ammonium chloride (10 mg; Vetnil, São Paulo, Brazil) was performed. Additionally, the horse showed a progressive weight loss due to chronic pain. In an attempt to improve patient quality of life, chemical neurolysis was applied with 1 mg of ammonium chloride perineurally to the palmar digital nerves at the abaxial sesamoid level. Long-term follow-up, two years post-injury, revealed lateral rotation of the limb’s axial axis leading to pure mechanical lameness, persistent grade 4/5 lameness (AAEP 2020), absence of pain on palpation, no localized swelling, and weight gain. Three years post-injury, the animal exhibits grade 2/5 (AAEP 2020) lameness, owing to natural remodeling, and retains full reproductive capability, demonstrating the ability to produce a healthy foal.

#### Case 2

The second case involved a 7-year-old mule weighing 238 kg, referred to the hospital, presenting with non-weight-bearing lameness (grade 5/5, AAEP 2020) of the left hindlimb. The animal had been involved in a vehicular accident on the same day. According to the owner, the incident occurred approximately three hours before hospital admission. On initial examination, the mule presented with a heart rate of 60 bpm, respiratory rate of 20 bpm, pink mucous membranes, capillary refill time of 2 s, and a rectal temperature of 37.1 °C. Soft tissue swelling and a skin wound on the dorsomedial aspect of the coronary band were observed. Manipulation of the limb revealed medial-to-lateral and dorsoplantar instability, rupture of the long digital extensor tendon, rupture of the medial collateral ligament, and joint capsule disruption. Internal joint structures were exposed; however, they were not in direct contact with the external environment. Upon wound exploration, communication with the DIPJ was confirmed.

**Fig. 3:**
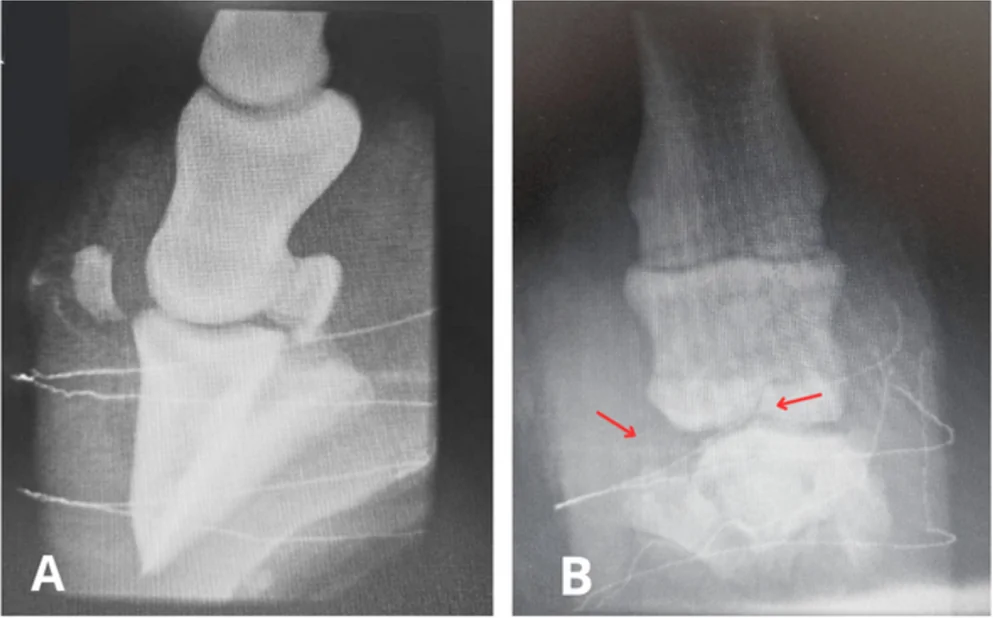
**A** Radiographic lateromedial view of the DIPJ with dorsal bone fragment measuring 1.13 cm in length. **B** Radiographic dorsopalmar view of the digit, showing absence of the third phalange proximal palmar process and linear fracture of the distal sesamoid bone

Radiographic examination revealed dorsomedial subluxation of the DIPJ and the presence of a 1.13 cm bone fragment dorsal to the joint, likely associated with avulsion of the medial collateral ligament, as well as a linear fracture in the distal sesamoid bone (Fig. 3).

**Fig. 4:**
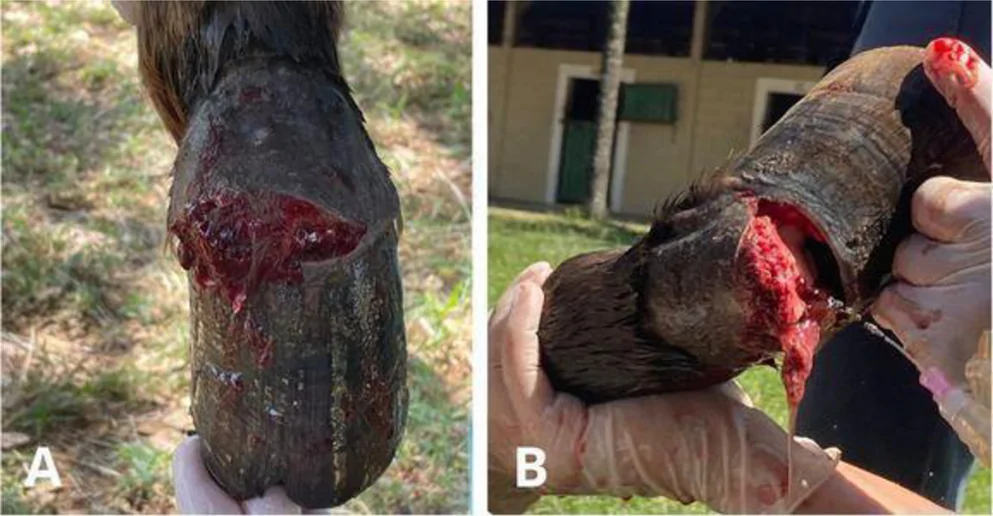
**A** Dorsal view of the digit, showing the dorsomedial wound in the coronary band area. **B** Dorsomedial view of the exposed luxated DIPJ under lavage procedure

The preanesthetic medication was administered with xylazine – 0.5 mg/kg, IV, and for total intravenous anesthesia were used ketamine (2.2 mg/kg, IV) and diazepam (0.05 mg/kg, IV). Intra-articular lavage was performed using lactated Ringer’s solution to remove debris, followed by debridement of damaged tissues while preserving the articular cartilage, including removal of the fragment part that showed signs of devitalization (Fig. 4). After joint manipulation and reduction, the skin at the coronary band was sutured using a simple interrupted pattern. Amikacin sulfate (150 mg) was administered intra-articularly, and intravenous regional limb perfusion (IRLP) was performed with ceftriaxone (2 g) diluted in 20 mL of physiological saline and lidocaine without vasoconstrictor (20 mL). A tourniquet was applied at the proximal metatarsus and maintained for 25 min. The limb was immobilized in a neutral weight-bearing position using a synthetic cast from the hoof to the proximal metatarsus, and the anesthetic recovery was assisted.

IRLP was repeated every 48 h for 7 days. Systemic treatment included gentamicin (6.6 mg/kg SID, IV) for 10 days, ceftiofur (5 mg/kg SID, IV) for 15 days, doxycycline (10 mg/kg BID, PO) for 7 days, flunixin meglumine (1.1 mg/kg SID, IV) for 3 days. The analgesic rescue was needed in this case 5 days after the intervention, was administred: phenylbutazone (4.4 mg/kg SID) for 7 days, and followed meloxicam (0.6 mg/Kg SID, PO; Ourofino, São Paulo, Brazil) for 7 days. A single dose of tetanus antitoxin (10,000 IU, SC; Lema Biologics, São Paulo, Brazil) was administered. In the postoperative time, the cast was daily monitored, in order to early detect signs of color change, odor, or any discharge. The severity of pain and the degree of limb weight-bearing during ambulation were meticulously monitored throughout the limb immobilization period.

**Fig. 5:**
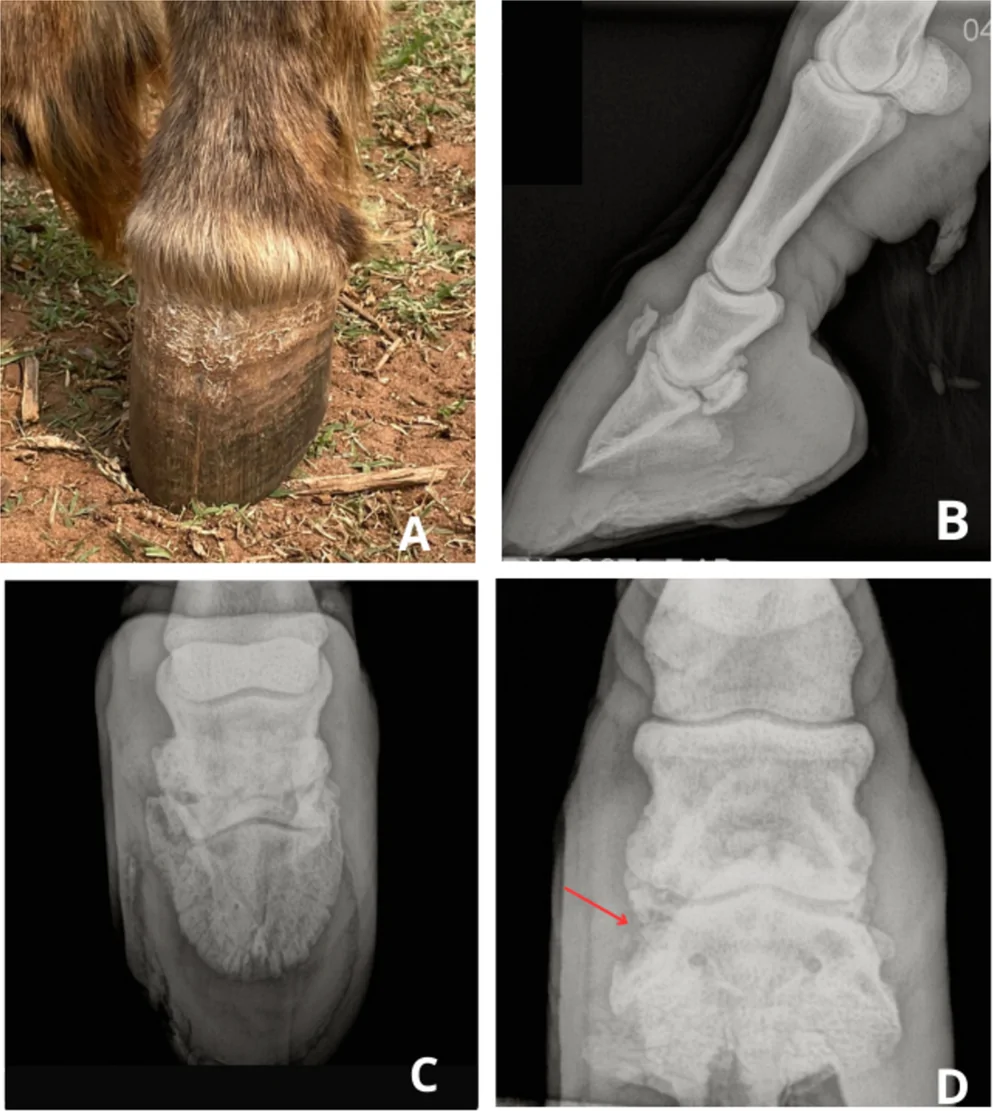
**A** Long-term follow-up of the left hindlimb showed no swelling or pain on palpation. **B** Lateromedial view: bone proliferation on the dorsal and plantar surface of the DIPJ, presence of the bone fragment dorsal to the DIPJ and reduced DIPJ space. **C** 60° dorsoproximal- plantarodistal oblique view: bone proliferation of the distal sesamoid bone. **D** Dorsoplantar view: consolidation of the third phalanx medial process and the distal sesamoid bone fracture

After 30 days, the cast was removed in the standing position under sedation (xylazine – 0.5 mg/kg, IV). The wound had completely healed, and the sutures were removed. The digit was cleaned, and a Robert Jones bandage was applied in combination with a 1 cm-thick polyvinyl chloride splint shaped to match the natural curvature of the limb, extending from the hoof to the proximal metatarsus along the dorsal surface. The animal presented with residual grade 2/5 lameness (AAEP 2020), and twice-daily controlled grazing was instituted. Mild local swelling and no pain on palpation were noted at this time. The mule was discharged after 45 days of hospitalization, and the splint remained in place on the property until 60 days post-treatment, with continued confinement recommended. During the long-term follow-up, two years after discharge, the animal presented with grade 2/5 lameness (AAEP 2020), with no signs of local swelling or pain on palpation (Fig. 5).

Radiographic examination revealed signs of DIPJ osteoarthritis, including bone irregularities on the articular surface and decreased joint space. Additionally, signs of proliferation and remodeling in the distal sesamoid bone. The mild phalanx fracture was fully consolidated, and the avulsive fragment of the third phalanx remained dorsal to the DIPJ (Fig. 5). Given the horse’s low activity and stable conditions, the owner chose not to pursue treatment for its osteoarthritis.

## Discussion

This report presents two uncommon cases of open DIPJ luxation, detailing clinical presentation, diagnosis, treatment, and long-term outcomes. In both cases, medial DIPJ luxation was observed at the time of evaluation. Although the animals had different injury histories, the medial direction of the dislocation can be explained by the lateral abduction and medial rotation of the joint upon ground impact during locomotion (Clayton 2010). The DIPJ contributes to 57% of the total axial rotation of the distal limb joints, thus playing a major role in distal limb displacement (Clayton 2010).

Donkeys have advantages in superficial wound healing due to a more efficient inflammatory response and a lower tendency for excessive granulation tissue formation. However, for complex injuries like distal interphalangeal (DIP) luxation, successful healing depends on a combination of anatomical traits, injury severity, overall health, and proper veterinary care (Herman 2009).

Given the involvement of the DIPJ and its exposure to the external environment in both cases, emergency treatment, including joint lavage to remove foreign material and microorganisms, debridement of contaminated and necrotic tissue, intravenous regional limb perfusion, and local and systemic antibiotic therapy, aimed to prevent septic arthritis, restore the intra-articular environment, and improve prognosis. Thereby maintaining the animal´s quality of life, as supported by Brogniez et al. (2012).

Despite its low prevalence in horses or donkeys, open joint luxation should be regarded as a surgical emergency due to its significant propensity to progress to severe infections, such as septic arthritis and osteomyelitis. In cases of systemic sepsis, euthanasia is often the outcome, as previously described by Honnas et al. (1992). Another consequence of DIPJ luxation is progressive cartilage degeneration, culminating in osteoarthritis due to tissue trauma and the inflammatory microenvironment. The limited regenerative capacity of articular cartilage makes treatment of such lesions challenging in the context of preserving joint function (van Zadelhoff et al. 2020). Osteoarthritis is a common complication of septic arthritis, as described by Reevs et al. (1989) and Brogniez et al. (2012). In humans, DIPJ osteoarthritis can occur due to dislocation alone; however, septic involvement significantly worsens the condition (Trickett et al. 2021).

Studies analyzing synovial fluid highlight the metabolic differences between septic and non-septic joints (Anderson et al. 2018, 2019). In septic arthritis, there is upregulation of catabolic mediators, such as matrix metalloproteinases, interleukin-1β, and tumor necrosis factor-alpha (Ehrle et al. 2015), resulting in more severe joint degeneration compared to non-septic cases. The greater extent and contamination of the lesion, longer exposure time, and direct contact of the joint with the ground were consistent with the more severe and prolonged lameness observed in Case 1. Additionally, the thoracic limbs bear approximately 60% of the equine body weight (Mitchell et al. 2014), which may contribute to the prolonged recovery period. Nevertheless, despite unfavorable prognostic factors, conservative treatment was successful in preserving life, albeit with residual lameness and preserving the reproductive capacity.

Whisenant et al. (2023) demonstrated that current treatment strategies for septic arthritis include systemic, intra-articular, and intravenous regional antibiotic therapy, as well as NSAIDs, joint lavage, debridement, and the use of drains via arthroscopy or arthrotomy. Anshul et al. (2023) reported the use of neurolytic agents, such as alcohol and phenol, for managing chronic pain. These agents act by degenerating nerve structures involved in nociception, thereby interrupting pain transmission and providing long-term analgesia.

The escalating threat of antimicrobial resistance is a profound global concern, particularly regarding the use of third-generation cephalosporins (Morton 2005). Even in the context of antibiotic resistance, the choice of ceftiofur as a first-choice antibiotic remains justified by the nature of synovial sepsis, which requires immediate, non-prodrug broad-spectrum bactericidal coverage. It is important to employ these potent antibiotics judiciously, strictly reserving them for life-threatening emergencies like synovial sepsis. The empirical use of ceftiofur is also justified by its targeted spectrum of activity, which effectively combats bacteria common in open wounds, given that the patients were found in unclean places, which create opportunities for infection (Pezzanite et al. 2022). Additionally, its synovial penetration and versatility allow its use for regional limb perfusion or intravenous administration (Pezzanite et al. 2022). During hospitalization, the antibiotic was not changed to reduce the patient’s pharmacological exposure.

Although surgical arthrodesis is described for DIPJ involvement, this technique presents limitations. Being a high-mobility joint located within the hoof capsule, it requires prolonged external immobilization, presents difficult access to articular extremities, and the anatomy and density of the distal phalanx make internal implant fixation particularly challenging (Honnas et al. 1992; Zubrod and Schneider 2005, 2019). Moreover, the hoof wall and internal laminae limit the use of dynamic compression plates to counteract the forces acting on the joint (Zubrod and Schneider 2019). In septic cases, the presence of internal implants may impair infection control, as the material provides a surface for biofilm formation (O’Toole et al. 2000), making spontaneous ankylosis a more viable option. Furthermore, no significant difference in time recovery was observed between conservative and surgical treatments (Heer et al. 2019).

While the metacarpophalangeal and metatarsophalangeal joints present similar mobility to the DIPJ, they show different prognoses with arthrodesis. In contrast to proximal interphalangeal arthrodesis—where return to athletic function may occur due to the joint’s low mobility—DIPJ arthrodesis typically results in permanent, non-painful lameness, allowing for use in breeding but not athletic activity. Due to the mobility of the joint, internal implants would be ideal, but their location within the hoof capsule limits surgical access (Nixon 2019). Ketzner et al. (2009) reported the use of casting for injuries of the pastern and hoof, and a combination of suturing and casting was also reported by McIlwraith et al. (2020). In the present report, conservative treatment of two DIPJ luxation cases proved to be successful.

The length of time the weight-bearing foot remains fully loaded appears to be a relevant limitation using the conservative treatment, including side effects on the contralateral limb, which would suffer greater pressure toward the limit standing position of these animals (Redden 2004), leading to more serious complications, such as laminitis. The mechanical stability in heavier horses will probably differ compared with a lighter horse, since the soft tissue would not maintain the joint in its anatomical form until tissue healing (Frisbie et al. 2020).

## Conclusion

Emergency care and prompt therapeutic intervention in cases of open DIPJ luxation are essential for successful management, allowing resolution without the need for facilitated ankylosis techniques or surgical arthrodesis. Although osteoarthritis may develop as a complication, conservative treatment was beneficial for the animal´s quality of life, allowing the animal to remain suitable for light work or reproductive purposes.

## Data Availability

No datasets were generated or analysed during the current study.

## References

[CR1] Anderson JR, Phelan MM, Clegg PD, Peffers MJ, Rubio-Martinez LM (2018) Synovial Fluid Metabolites Differentiate between Septic and Nonseptic Joint Pathologies. J Proteome Res 17(8):2735–2743. 10.1021/acs.jproteome.8b00190 PMC6092013 29969035

[CR2] Anderson JR, Smagul A, Simpson D, Clegg PD, Rubio-Martinez LM, Peffers MJ (2019) The synovial fluid proteome differentiates between septic and nonseptic articular pathologies. J Proteom 202103370. 10.1016/j.jprot.2019.04.020 PMC6549134 31028944

[CR3] Anshul, Malhotra N, Kumar A, Rohilla J, Sinha K N (2023) Comparative Evaluation of Intraarticular Facet Joint Injection Versus Medial Branch Block in Patients With Low Back Pain: A Randomised Controlled Study. Cureus 15(11):e49232. 10.7759/cureus.49232 PMC10746869 38143628

[CR4] Balaam TM, Miller S (2007) Severe open metacarpophalangeal joint luxation in a mule foal. Equine Vet Educ 19(10):528–531. 10.2746/095777307X229309

[CR5] Borges LPB, dos Santos GMA, Oliveira RA, da Silva LO, de Souza LA, Perdigao HH, Guimaraes MM, Silveira JAS, Duarte MD, Teixeira PPM (2020) Metacarpophalangeal joint luxation with joint capsule rupture and bone exposure in a horse. Vet Med 65(7):309–313. 10.17221/170/2019-VETMED

[CR6] Brogniez L, Launois T, Perrin R, Horn L, Clegg P, Coomer R, Gabriel A, Mesnil S, Carstanjen B, Desbrosse F, Kirschvink N, Vandeweerd JM (2012) Treatment of a severe distal forelimb wound presenting with extensive laceration and distal interphalangeal joint luxation in a donkey. Pferdeheilkunde 28(2): 160–166. https://pferdeheilkunde.de/files/20120204.pdf. Accessed 31 Jan 2026

[CR7] Chan CC, Murphy H, Munroe GA (2000) Treatment of chronic digital septic tenosynovitis in 12 horses by modified open annular ligament desmotomy and passive open drainage. Vet Rec 147(14):388–393. 10.1136/vr.147.14.388 11073001

[CR8] Clayton HM (2010) Biomechanics of the distal interphalangeal joint. J Equine Vet Sci 30(8):401–405. 10.1016/j.jevs.2010.07.007

[CR9] de Souza TC, Burford J, Busschers E, Freeman S, Suthers JM (2024) Multicenter study investigating long-term survival after synovial lavage of contaminated and septic synovial structures in horses presented to 10 UK referral hospitals. Vet Surg 53(6):1083–1092. 10.1111/vsu.14107 38863154

[CR10] Ehrle A, Lischer CJ, Lasarzik J, Einspanier R, Bondzio A (2015) Synovial Fluid and Serum Concentrations of Interleukin-1 Receptor Antagonist and Interleukin-1ß in Naturally Occurring Equine Osteoarthritis and Septic Arthritis. J Equine Vet Sci 35(10):815–822. 10.1016/j.jevs.2015.07.023

[CR11] Fraser BSL, Bladon BM (2004) Tenoscopic surgery for treatment of lacerations of the digital flexor tendon sheath. Equine Vet J 36(6):528–531. 10.2746/0425164044877396 15460078

[CR12] Frisbie DD, Johnson SA (2020) Medical treatment in joint disease. In: Baxter GM (ed) Adams and Stashak’s Lameness in Horses, 7th edn. Wiley-Blackwell, pp 1348–1363

[CR14] Heer C, Fürst AE, Chicca FD, Jackson MA (2019) Comparison of 3D-assisted surgery and conservative methods for treatment of type III fractures of the distal phalanx in horses. Equine Veterinary Educ 32:42–51. 10.1111/eve.13232

[CR15] Herman CL (2009) The Anatomical Differences between the Donkey and the Horse. In: Matthews N.S. and Taylor T.S. (eds.) Veterinary Care of Donkeys, International Veterinary Information Service, Ithaca NY. Accessed 31 Jan 2026

[CR16] Honnas CM, Acvs D, Welch RD, Ford TS, Vacek JR, Watkins JP (1992) Septic Arthritis of the Distal Interphalangeal Joint in 12 Horses. Vet Surg 21(4):261–268. 10.1111/j.1532-950x.1992.tb00061.x 1455633

[CR17] Janicek JC, Keegan KG (2007) What is your diagnosis? Subluxation of DIPJ. J Am Vet Med Assoc 230(9):1307–1308. 10.2460/javma.230.9.1307 17472553

[CR18] Ketzner KM, Stewart AA, Byron CR, Stewart M, Gaughan EM, Vanharreveld PD, Lillich JD (2009) Wounds of the pastern and foot region managed with phalangeal casts: 50 cases in 49 horses (1995–2006). Aust Vet J 87(9):363–368. 10.1111/j.1751-0813.2009.00471.x 19703138

[CR19] Lescun TB, Morisset SM, Fugaro MN, Blevins WE (2004) Facilitated ankylosis of the distal interphalangeal joint in a foal. Aust Vet J 82(5):282–285. 10.1111/j.1751-0813.2004.tb12704.x 15181928

[CR20] McIlwraith CW, Kawcak C, Baxter GM, Goodrich LR, Valberg SJ (2020) Principles of Musculoskeletal Disease. In: Baxter GM (ed) Adams and Stashak’s Lameness in Horses, 7th edn. Wiley-Blackwell, pp 801–874

[CR21] Mitchell CF, Fugler LA, Eades SC (2014) The management of equine acute laminitis. Vet Med (Auckl) 6:39–47. 10.2147/VMRR.S39967 PMC6067769 30101095

[CR22] Morton AJ (2005) Diagnosis and treatment of septic arthritis. The Veterinary clinics of North America. Equine Pract 21(3):627–vi. 10.1016/j.cveq.2005.08.001 16297725

[CR23] Nixon AJ, Ducharme NG, Bertone AL (2019) Fractures of the Distal Phalanx. In: Nixon AJ (ed) Equine Fracture Repair, 2nd edn. Wiley Online Library, pp 219–241. 10.1002/9781119108757.ch14

[CR25] O’Toole G, Kaplan HB, Kolter R (2000) Biofilm formation as microbial development. Annu Rev Microbiol 54:49–79. 10.1146/annurev.micro.54.1.49 11018124

[CR26] Pezzanite LM, Hendrickson DA, Dow S, Stoneback J, Chow L, Krause D, Goodrich L (2022) Intra-articular administration of antibiotics in horses: Justifications, risks, reconsideration of use and outcomes. Equine Vet J 54(1):24–38. 10.1111/evj.13502 34459027

[CR27] Pille F, Martens A, Oosterlinck M, Dumoulin M, Dewulf J, Gasthuys F (2009) A retrospective study on 195 horses with contaminated and infected synovial cavities. Vlaams Diergeneeskd Tijdschr 2(78):97–104. 10.21825/vdt.87503

[CR28] Raayat Jahromi A, Vajdi N (2017) Successful Management of Severe Open Metacarpophalangeal Joint Dorsal Luxation in a Horse. J Equine Vet Sci 1(48):48–51. 10.1016/j.jevs.2016.08.011

[CR29] Reevs MJ, Yovich JV, Turner AS (1989) Miscellaneous Conditions of the Equine Foot. Vet Clin North Am Equine Pract 1(5):221–242. 10.1016/S0749-0739(17)30612-0 2565157

[CR30] Redden RF (2004) Preventing laminitis in the contralateral limb of horses with nonweight-bearing lameness. Clin Techniques Equine Pract 3(1):57–63. 10.1053/j.ctep.2004.07.005

[CR31] Trickett RW, Brock J, Shewring DJ (2021) The non-operative management of bony mallet injuries. J Hand Surg Eur 46(5):460–465. 10.1177/1753193421992986 33588631

[CR32] van Zadelhoff C, Schwarz T, Smith S, Engerand A, Taylor S (2020) Identification of Naturally Occurring Cartilage Damage in the Equine Distal Interphalangeal Joint Using Low-Field Magnetic Resonance Imaging and Magnetic Resonance Arthrography. Front Vet Sci 6508. 10.3389/fvets.2019.00508 PMC6999043 32064268

[CR33] Whisenant KD, Ruggles AJ, Stefanovski D, Woodie JB, Hopper SA, Embertson RM (2023) Prognosis for survival to discharge and racing performance in Thoroughbred foals treated for single joint septic arthritis (2009–2016). Equine Vet J 55(4):607–617. 10.1111/evj.13892 36210723

[CR34] Wright IM, Smith MRW, Humphrey DJ, Eaton-Evans TCJ, Hillyer MH (2003) Endoscopic surgery in the treatment of contaminated and infected synovialtrickett cavities. Equine Vet J 35(6):613–619. 10.2746/042516403775467225 14515964

[CR35] Yovich JV, Turner AS, Stashak TS, McIlwraith CW (1987) Luxation of the metacarpophalangeal and metatarsophalangeal joints in horses. Equine Vet J 19(4):295–298. 10.1111/j.2042-3306.1987.tb01414.x 3622457

[CR36] Zubrod CJ, Schneider RK (2005) Arthrodesis techniques in horses. Vet Clin North Am Equine Pract 3(21):691–711. 10.1016/j.cveq.2005.07.004 16297728

[CR37] Zubrod CJ, Schneider RK (2019) Arthrodesis of the distal Interphalangeal Joint. In: Nixon AJ (ed) Equine Fracture Repair, 2nd edn. Wiley Online Library, pp 257–263. 10.1002/9781119108757.ch16

